# Development of male breast cancer in a patient with prostate cancer during androgen deprivation therapy

**DOI:** 10.1002/iju5.12409

**Published:** 2022-01-11

**Authors:** Hiroya Mizusawa, Akira Komatsu, Yuji Mimura, Toshitaka Maejima

**Affiliations:** ^1^ Department of Urology National Hospital Organization Shinshu Ueda Medical Center Ueda Nagano Japan; ^2^ Department of Breast Surgery National Hospital Organization Shinshu Ueda Medical Center Ueda Nagano Japan; ^3^ Department of Pathology and Laboratory Medicine National Hospital Organization Shinshu Ueda Medical Center Ueda Nagano Japan

**Keywords:** adverse effect, double cancer, estrogen preparation, gynecomastia, hormone therapy

## Abstract

**Introduction:**

Male breast cancer accounts for <1% of all breast cancer. We report a patient with prostate cancer during hormone therapy who developed breast cancer.

**Case presentation:**

An 88‐year‐old male underwent androgen deprivation therapy for prostate cancer and developed an induration in the left breast 7 years after the start of treatment. After close examination, he was diagnosed with left breast cancer with lymph node metastasis. The prostate cancer was stable in a hormone‐sensitive state. Left mastectomy was performed and the pathological diagnosis was invasive ductal carcinoma.

**Discussion:**

In addition to our patient, seven patients who developed breast cancer during hormone therapy for prostate cancer were examined. Five of six patients had stage II or above, and four patients had lymph node metastases. Although local breast symptoms are frequently observed as adverse effects of hormone therapy, caution is warranted regarding male breast cancer.

**Abbreviations & Acronyms**
ER: estrogen receptor
Her2: human epidermal growth factor receptor‐related 2
PGR: progesterone receptor
PSA: prostate‐specific antigen

**Keynote message**

We report a patient with prostate cancer who developed breast cancer during hormone therapy. Seven similar cases were previously reported. Most were advanced breast cancer. Caution should be exercised in the event of local breast symptoms.

## Introduction

Male breast cancer accounts for <1% of all breast cancer.^1^ We report a patient with prostate cancer who developed breast cancer during androgen deprivation therapy. We also examined similar previously reported cases.

## Case presentation

**Fig. 1:**
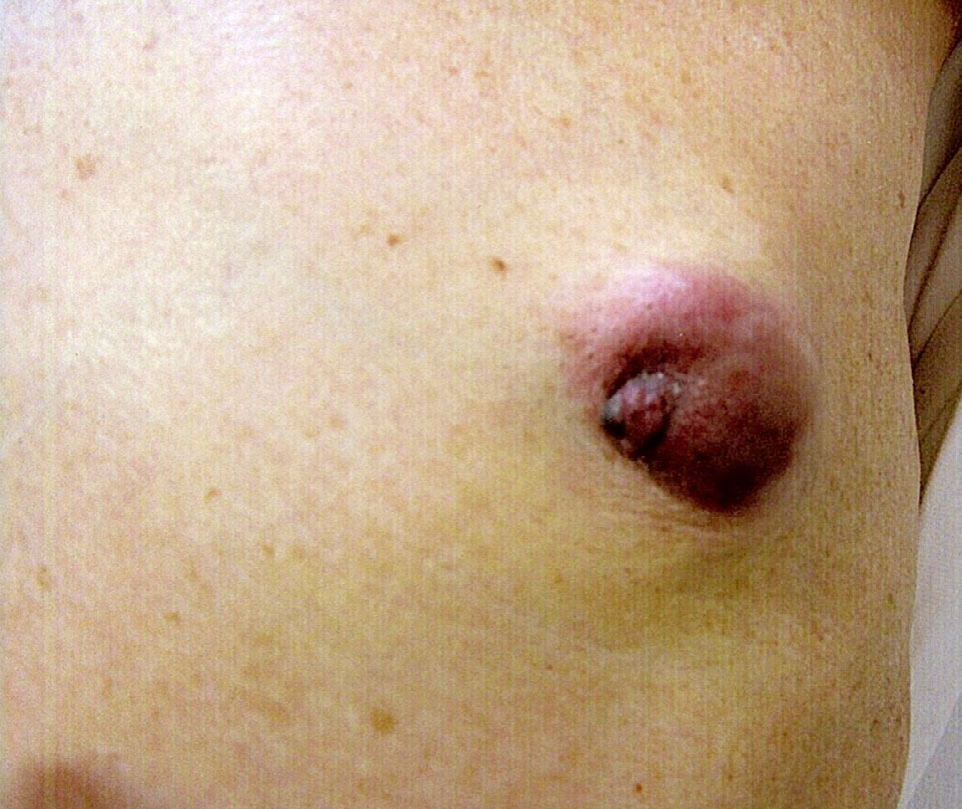
Appearance of the left breast. Gynecomastia and induration were observed.

**Fig. 2:**
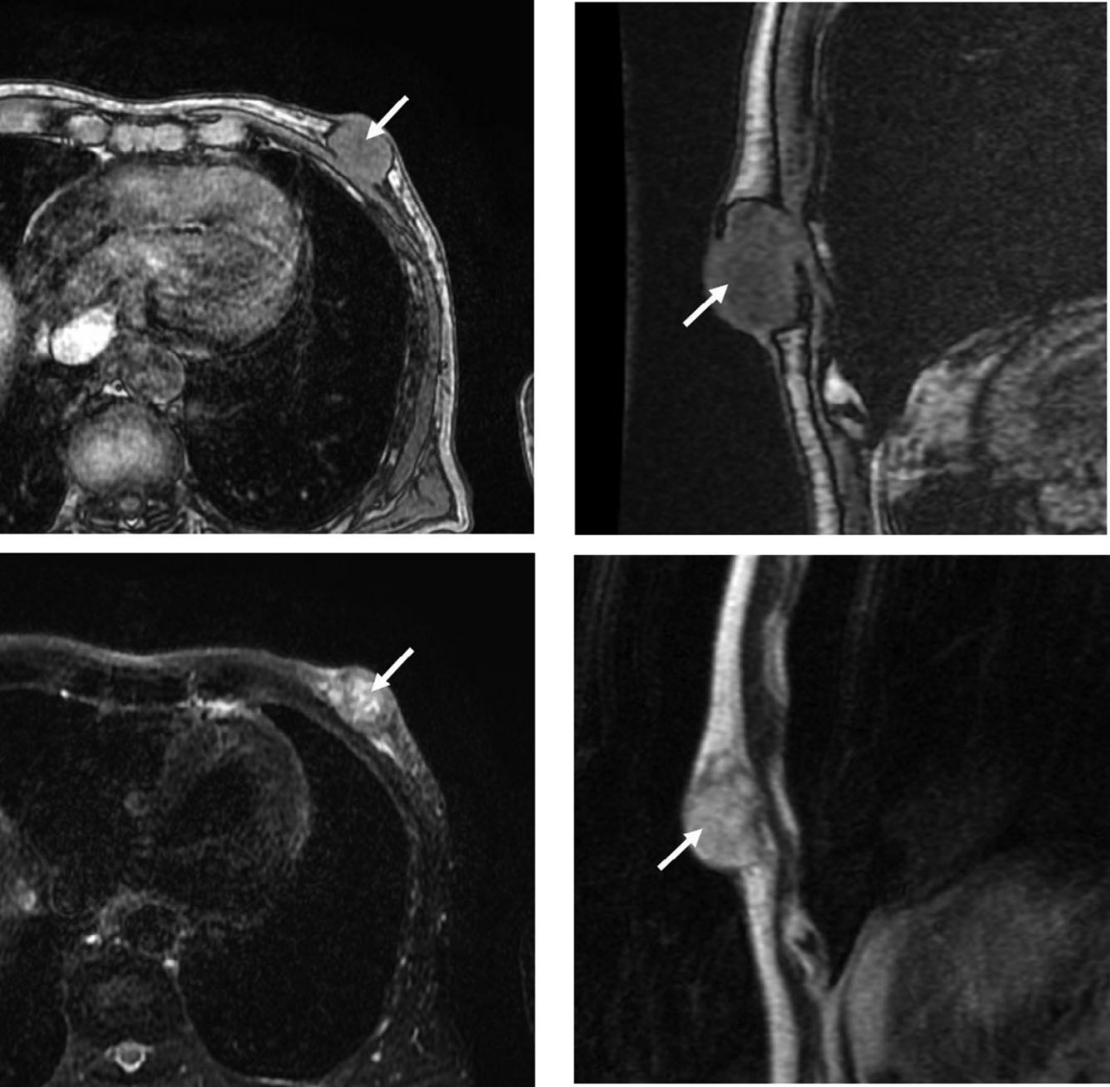
Simple magnetic resonance imaging of the left breast. Tumors with unclear boundaries and inhomogeneous content (arrows). Upper: T1‐weighted image (left: transverse section, right: sagittal section). Lower: T2‐weighted image (left: transverse section, right: sagittal section).

**Fig. 3:**
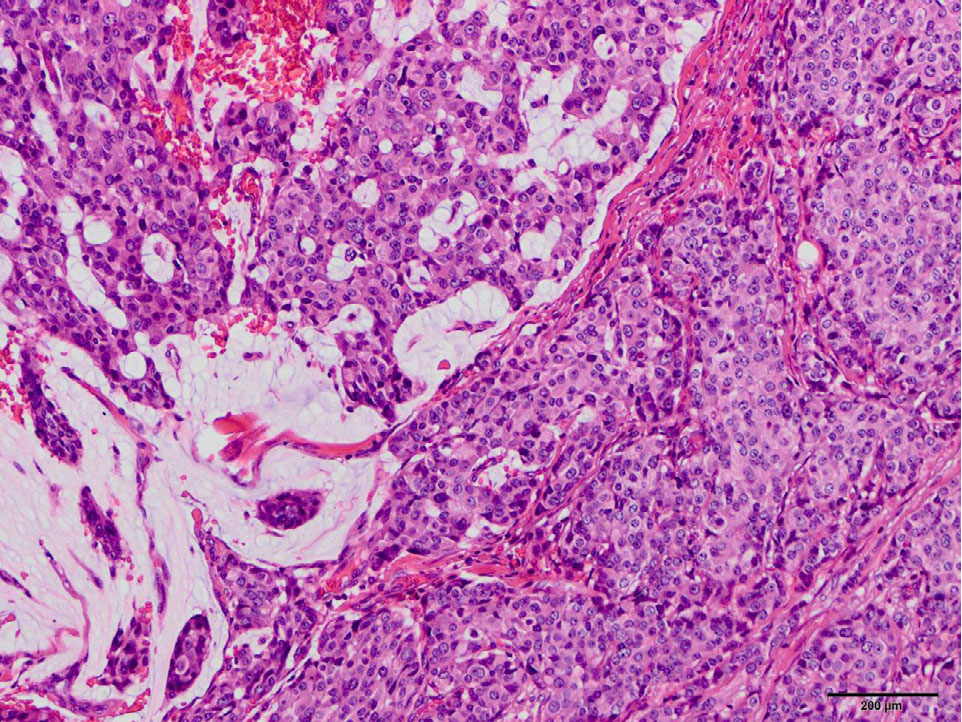
Microscopic appearance of the left total mastectomy specimen. Mainly invasive ductal carcinoma accompanied by apocrine metaplasia. Expansive growth with eosinophilic and granular changes was observed in the vesicles. The bar length is 100 μm. Hematoxylin and eosin staining.

An 80‐year‐old male was referred to our hospital by his attending doctor because of a high PSA level (9.1 ng/mL). He had a history of cholecystectomy for gallstones. His family history was non‐contributory, except for gastric cancer in his brother. He had no specific urinary symptoms. After prostate biopsy, he was diagnosed with adenocarcinoma of Gleason score 3 + 3 (T1cN0M0). Thus, androgen deprivation therapy was performed with bicalutamide and leuprorelin acetate. Seven years after the start of hormone therapy, a soybean‐sized induration with mild pain was found in the left breast. Subsequently, the pain disappeared and the patient was followed up. One year and 8 months later, the pain recurred in the left breast. The breast mass had grown to the size of a thumb tip, exhibiting mild tenderness (Fig. 1). The PSA level was stable at as low as 0.357 ng/mL. The patient underwent magnetic resonance imaging, revealing a lobulated mass of 36 × 29 × 21 mm in size with an unclear boundary in the left chest wall (Fig. 2). He was diagnosed with left breast cancer (T4N1M0, stage IIIB) in the breast surgery department, and underwent left mastectomy, left axillary lymph node dissection, and skin grafting at a nearby hospital. Pathologically, he was diagnosed with invasive ductal carcinoma, s, ly1, v1, n (+), ER (+) 100%, PGR (+) 70%, Her2/neu, Score [1+], PSA(−) (Fig. 3). Considering his high age, he received tamoxifen for 2 years. Subsequently, he was followed up for another 3 years without recurrence or metastasis. Eight years after the surgery for breast cancer, lung/liver metastases of breast cancer, lymph node recurrence, and cancerous pleurisy were noted, and tamoxifen treatment was resumed. For the prostate cancer, hormone therapy was continued and the PSA gradually increased to 3.985 ng/mL after admission. Following the wishes of the patient and his family, the prostate and breast cancers were treated by an attending doctor.

## Discussion

Male breast cancer accounts for <1% of all breast cancer and <0.1% of cancer‐related deaths in males. Compared with breast cancer in females, male breast cancer is often diagnosed later, when they are in their 60s, and is typically diagnosed at an advanced stage. Risk factors for male breast cancer include a family history of breast and ovarian cancer, BRCA2 mutation, Klinefelter syndrome, old age, obesity, use of estrogen preparations, and exposure to radiation.^1^, ^2^, ^3^ In our case, the patient’s age was a risk factor.

A total of seven male breast cancer cases diagnosed during hormone therapy for prostate cancer have been reported to date.^4^, ^5^, ^6^, ^7^, ^8^, ^9^, ^10^ These reported cases in addition to our own case discussed in the present study are summarized in Table 1. The median age of the patients was 75 years and the median duration of hormone therapy was 33 months. All patients underwent either castration or treatment with a luteinizing hormone‐releasing hormone analogue, and all except one patient received either estrogen or antiandrogen preparations.

**Table 1:** Reported cases of prostate cancer with breast cancer developing during hormone therapy in Japan

	*n* = 8
Age	75 (58–88)
Tumor side	
Left	5/8
Right	1/8
Bilateral	2/8
Initial symptom	
Induration	6/8
Pain	3/8
Bleeding	2/8
Tumor size	
<2 cm	1/6
≥2 cm, <5 cm	3/6
≥5 cm	2/6
Breast cancer stage	
I	1/6
II	3/6
III	2/6
Prostate cancer status	
Hormone‐sensitive	5/7
Castration‐resistant	2/7
Hormone therapy	
Castration alone	1/8
Castration + estrogen (+antiandrogens)	4/8
Castration + antiandrogen(s)	3/8
Duration of hormone therapy	
<2 years	2/8
≥2 years, <5 years	5/8
≥5 years	1/8
Immunostaining‐positive	
ER	5/6
PGR	5/7
Her2	2/5
PSA	2/6

Local symptoms in the breast are a common complication of hormone therapy for prostate cancer. All hormone preparations that were used in the eight cases are known to have side effects that include gynecomastia and breast engorgement, with the incidence ranging from 13% to 16% for leuprorelin, 40% to 77% for fosfestrol, and 16% to 79% for flutamide.^11^

Awareness of male breast cancer is relatively low among individuals, with few opportunities for regular testing. Male breast cancer is also often neglected by physicians and typically diagnosed at an advanced stage.^2^ This issue is particularly important for prostate cancer patients because urologists involved in the treatment of prostate cancer may empirically regard breast symptoms as adverse effects of hormone therapy. Among the eight cases identified in the present study, lymph node metastasis was found in four of six that described disease staging, whereas one of the two cases without the description of disease staging had an enlarged mass exceeding 5 cm at the time of breast cancer diagnosis. These findings indicate that at least six cases were stage II or above, and that three were stage IIB or above.

Breast symptoms as side effects of hormone therapy typically develop early in the treatment course. Thus, physicians should suspect breast cancer when delayed breast enlargement is observed, and should follow‐up with magnetic resonance imaging and/or with a breast surgeon as needed.

Surgery was performed for breast cancer in all eight cases examined in the present study. On pathology, all were diagnosed as invasive ductal carcinoma, which is characteristic of male breast cancer.^2^ PSA immunostaining was performed in six cases, of which two were positive. A low level of PSA expression has been described in organs other than the prostate such as the mammary gland. As such, studies suggest that PSA positivity cannot be used to discriminate metastatic breast cancer from primary breast cancer.^12^, ^13^ In our case, we diagnosed the patient with primary breast cancer because prostate cancer was considered to be controlled with hormone therapy. Male breast cancer is often characterized by the expression of hormone receptors; specifically, over 90% of all cases are ER‐positive and over 80% are PGR‐positive.^1^, ^2^, ^3^ This trend was also observed in the eight cases we examined in the present study.

The use of estrogen preparations is considered one of the risk factors for male breast cancer. However, the use of estrogen preparations as hormone therapy for prostate cancer has markedly decreased in recent years. It is highly unlikely that the current hormone therapy regimens increase the risk of male breast cancer. In addition, active surveillance should be considered as the first option in low‐risk cases such as ours.

Pathogenic variants are commonly found in male breast cancer patients and the National Comprehensive Cancer Network guidelines recommend genetic testing for this patient population.^14^ Individuals with a family history of female cancers are considered to have a high risk of male breast cancer; therefore, appropriate information and close monitoring should be provided to such individuals. Genetic testing is also recommended for prostate cancer patients with metastasis,^14^ highlighting the need to increase awareness about male breast cancer among urologists.

## Conclusion

We reported a case of breast cancer that developed during hormone therapy for prostate cancer. Caution should be exercised in the event of local breast symptoms and a specialist should be consulted immediately if abnormalities are observed.

## Author Contributions

**Hiroya Mizusawa:** Conceptualization; Writing – original draft. **Akira Komatsu:** Data curation; Supervision; Writing – review & editing. **Yuji Mimura:** Supervision; Writing – original draft; Writing – review & editing. **Toshitaka Maejima:** Supervision; Writing – review & editing.

## Conflict of interest

The authors declare no conflict of interest.

## Approval of the research protocol by an Institutional Reviewer Board

Not applicable.

## Informed consent

Not applicable.

## Registry and the Registration No. of the study/trial

Not applicable.
